# How Do We Talk With People Living With Dementia About Future Care: A Scoping Review

**DOI:** 10.3389/fpsyg.2022.849100

**Published:** 2022-04-12

**Authors:** Mandy Visser, Hanneke J. A. Smaling, Deborah Parker, Jenny T. van der Steen

**Affiliations:** ^1^Department of Public Health and Primary Care, Leiden University Medical Center, Leiden, Netherlands; ^2^University Network for the Care Sector Zuid-Holland, Leiden University Medical Center, Leiden, Netherlands; ^3^Improving Palliative, Aged and Chronic Care Through Clinical Research and Translation (IMPACCT), Faculty of Health, University of Technology Sydney, Ultimo, NSW, Australia; ^4^Department of Primary and Community Care, Radboud University Medical Center, Nijmegen, Netherlands

**Keywords:** dementia, advance care planning, communication, scoping review, palliative aged care

## Abstract

A diagnosis of dementia often comes with difficulties in understanding a conversational context and expressing how one feels. So far, research on how to facilitate advance care planning (ACP) for people with dementia focused on defining relevant themes and topics for conversations, or on how to formalize decisions made by surrogate decision makers, e.g., family members. The aim of this review is to provide a better scope of the existing research on practical communication aspects related to dementia in ACP conversations. In November 2020, seven databases were searched to select papers for inclusion (Proquest, Medline, Embase, Scopus, Psycinfo, Amed, and Cinahl). This search was updated in December 2021. The search strategy consisted of three tiers (related terms to “dementia,” “communication” and “ACP”), intersected by using the Boolean term “AND,” and resulted in 787 studies. Two researchers followed explicit criteria for two sequential levels of screening, based on titles and abstracts and full papers. A total of 22 studies were included for data analysis. Seven topics (i.e., importance of having ACP conversations, knowledge gap, inclusion of people with dementia in ACP conversations, policy vs. practice, adapting to cognitive changes, adapting to psychosocial changes, and adapting to emotional changes) emerged clustered around two themes (i.e., communicating with people with dementia in ACP, and changes in communication due to dementia). This scoping review provides practical suggestions for healthcare professionals to improve ACP communication and uncovered gaps in research on communication aspects related to dementia in ACP conversations, such as non-verbal behavior, timing and implementation, and personal preferences.

## Introduction

Advance care planning (ACP) can be described as the process that supports adults at any age or stage of health in understanding and sharing their personal values, life goals and preferences regarding medical care (Sudore et al., 2017). Performing ACP in early stages of any disease potentially increases the likelihood that a patient’s wishes will be incorporated into care decisions, and reduce unwanted hospitalization and intensive treatments at the end of life [Detering et al., 2010; Silveira et al., 2010; for a systematic review on effects of ACP on end-of-life care, see Brinkman-Stoppelenburg et al. (2014)]. ACP has been associated with a range of positive end-of-life outcomes. Although the evidence base is still limited, this could be especially relevant for people with dementia, as they are likely to experience a prolonged period of cognitive decline, starting in early stages of the disease trajectory [for a systematic review on effects of ACP in improving end-of-life outcomes for people with dementia, see Dixon et al. (2018)].

Changes in cognitive abilities caused by dementia may not only impact decision-making capacities, but are also likely to affect the ability to process information and to participate in conversations. Difficulties in understanding a conversational context and expressing feelings may lead to several communication challenges, depending on the dementia condition (Rousseaux et al., 2010; Visser et al., 2020). For people with dementia, a simple chat with family members can be difficult, let alone having conversations with healthcare professionals about ACP. As a result, it is not very common for healthcare professionals to initiate ACP conversations with people with dementia (Azizi et al., 2022), and if any important ACP topics need to be discussed, they gradually turn to a proxy decision maker, like a family caregiver. However, people with dementia should have an active role as long as possible as well, when planning their future care (Van den Block, 2019). Healthcare professionals may adapt their communication strategies in ACP conversations to the cognitive abilities of people with dementia.

So far, research on how to facilitate ACP for people with dementia specifically focuses on defining relevant themes and topics for conversations, or on how to formalize decisions made by surrogates (Ampe et al., 2016; Tilburgs et al., 2018). As Van den Block (2019) describes in her editorial on the current state of research on ACP for people with dementia, much of the existing literature emphasizes on answering questions concerning the “what” of ACP, rather than understanding the “how” of ACP communication. Questions such as “How do we overcome the barriers for ACP conversations with people with dementia?” and “How to tailor communication in order to facilitate ACP conversations with people with dementia?” are still to be answered. A better scope of the existing research on communication aspects related to dementia in ACP conversations is needed to uncover any gaps in research in order to formulate communication strategies for healthcare professionals to improve their ACP practices, impacting the lives of people with dementia and their family members.

The aim of this scoping review is to examine how communication difficulties related to dementia are addressed in the literature (following the explicit scoping review method by Arksey and O’Malley, 2005). This paper serves as a starting point for future research and clinical practice, highlighting the contribution of involving people with dementia in making decisions and plans around their care.

## Methods

### Eligibility Criteria

The study involved original empirical research papers focusing on communication and conversations about ACP or future palliative care, excluding discussions of immediate care relief. Subjects in these studies were to be people with dementia, family caregivers and/or healthcare professionals caring for people with dementia. Following scoping methods, no papers were excluded based on quality assessments of design and analyses, and no meta-analyses were used to aggregate findings (Arksey and O’Malley, 2005).

### Information Sources and Search

In November 2020, a total of seven databases were searched to select papers for inclusion (Proquest, Medline, Embase, Scopus, Psycinfo, Amed and Cinahl). This search was updated in December 2021. Three tiers of the search strategy (related terms to “dementia,” “communication” and “ACP”) were intersected by using the Boolean term “AND,” as presented in Table 1.

**TABLE 1:** Overview search strategy.

Tier 1^1^	AND	Tier 2	AND	Tier 3
Dementia OR		Communicate OR		Advance care planning OR
Alzheimer’s disease OR		Social OR		Palliative
Frontotemporal dementia OR		Conversation		
Vascular dementia OR				
Lewy body disease OR				
Parkinson’s disease OR				
Cognitive impair				

*^1^Tier 1 is based on the most common forms of dementia according to Alzheimer’s Association (https://www.alz.org/alzheimers-dementia/what-is-dementia/types-of-dementia): Alzheimer’s disease, frontotemporal dementia, vascular dementia, Lewy body disease, Parkinson’s disease.*

### Paper Screening, Extraction and Analysis

Two sequential levels of screening were undertaken independently by two researchers (MV and HS), based on: (1) titles and abstracts; and (2) full-text papers. Any disagreement was reconciled by consensus. Inclusion criteria were as follows: (1) papers must describe original empirical research; (2) involve human subjects that have been diagnosed with dementia or care for people diagnosed with dementia; (3) include communication aspects related to dementia in either variables or outcomes, and (4) study conversations around future palliative care or ACP. Papers were excluded (1) if published in a language other than English, (2) if the study focused on nursing homes residents or older people in general (with no results reported on an identifiable subgroup of people with dementia). and (3) focused on discussions of immediate care relief. An initial data extraction was conducted by MV, focusing on type of research, participants, setting, and aims of the papers. Following this, two researchers (MV and JS) independently performed an initial content analysis to map out potential topics related to the aim of this research. The research team (MV, HS, JS, and DP) engaged in a iterative process of discussing findings related to the topics to enhance analytical rigor and achieve consensus on outcomes.

## Results

### Search Results and Study Characteristics

**FIGURE 1:**
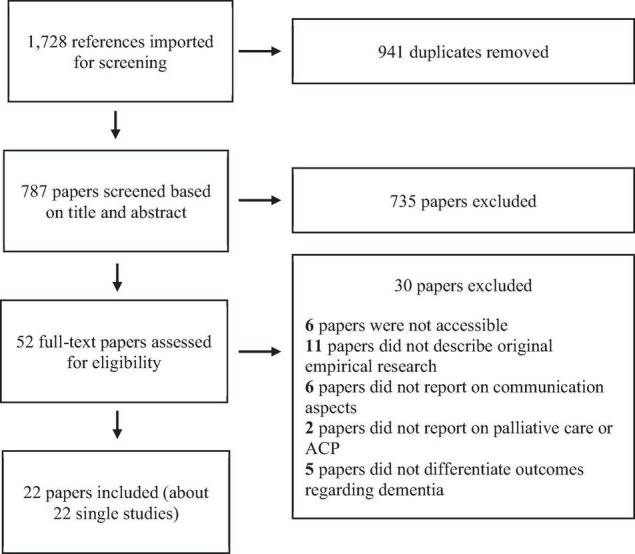
PRISMA flowchart procedure paper screening.

As illustrated in Figure 1, our search strategy resulted in 1,728 references. After removing duplicates, 756 references were imported to Covidence systematic review software (Veritas Health Innovation, Australia) to manage abstract- and full paper screening. After the title and abstract screening, 735 of 787 papers were excluded as they did not met all inclusion criteria as described above; in the full-text screening, 30 out of 52 papers were excluded for several reasons [i.e., not being accessible (6), or not describing original empirical research (11), not reporting on communication aspects (6), or palliative care or ACP (2) and not reporting dementia specific outcomes (5), leaving 22 papers for analysis].

As listed in Table 2, of the 22 included papers, 13 were published since 2016 (Ampe et al., 2016, 2017; Morin et al., 2016; Sinclair et al., 2016; Aasmul et al., 2018; Givens et al., 2018; Hill et al., 2018; Tilburgs et al., 2018; de Vries and Drury-Ruddlesden, 2019; Goossens et al., 2020a,b; Sussman et al., 2020; Yeh et al., 2021). The majority of papers described qualitative studies (*n* = 14), using methods such as interviews, focus groups and ethnographic observations (Godwin and Waters, 2009; Johnson et al., 2009; Sims-Gould et al., 2010; Goodman et al., 2013; Poppe et al., 2013; Beernaert et al., 2014; Ampe et al., 2016; Givens et al., 2018; Hill et al., 2018; Tilburgs et al., 2018; de Vries and Drury-Ruddlesden, 2019; Goossens et al., 2020a; Sussman et al., 2020). Only three papers described quantitative methods; two cluster randomized controlled trials (Ampe et al., 2017; Goossens et al., 2020b) and one survey described a single survey (Morin et al., 2016). Two studies used a mixed methods approach (Karel et al., 2007; Yeh et al., 2021) and two Delphi studies were included (van der Steen et al., 2014; Sinclair et al., 2016; Sussman et al., 2020). Papers described studies that were conducted in ten different countries, with five studies conducted in Belgium (Beernaert et al., 2014; Ampe et al., 2016, 2017; Goossens et al., 2020a,b), four in the United Kingdom (Godwin and Waters, 2009; Goodman et al., 2013; Poppe et al., 2013; Sinclair et al., 2016) and four in the United States of America (Kayser-Jones, 2002; Karel et al., 2007; Givens et al., 2018; Yeh et al., 2021). Other studies were conducted in Canada (Sims-Gould et al., 2010; Hill et al., 2018; Sussman et al., 2020), Norway (Aasmul et al., 2018), France (Morin et al., 2016), Netherlands (Tilburgs et al., 2018), Australia (Johnson et al., 2009) and New Zealand (de Vries and Drury-Ruddlesden, 2019). One paper used Delphi study methodologies including different countries across the world (van der Steen et al., 2014).

**TABLE 2:** Papers included.

Author (et al.)	Published	Title	Country	Type	Population, sample
Aasmul et al.	2018	Advance Care Planning in Nursing Homes—Improving the Communication Among Patient, Family, and Staff: Results From a Cluster Randomized Controlled Trial (COSMOS)	Norway	Quant*, CRCT*	545 residents, nursing home
		*Aim: investigate effect of ACP on communication among NH staff, patient, and family and nursing staff distress*			
Ampe et al.	2016	Advance care planning for nursing home residents with dementia: policy vs. practice	Belgium	Qual*, observations	20 nursing homes
		*Aim: evaluate ACP policy in nursing homes and the involvement of residents and families, and how policy relates to practice*			
Ampe et al.	2017	Advance care planning for nursing home residents with dementia: Influence of “we DECide” on policy and practice	Belgium	Quant*, Pre-test-post-test CRCT*	90 care workers, 18 dementia care units
		*Aim: pilot ACP intervention in terms of influence on ACP policy and practice in nursing homes*			
Beernaert et al.	2014	Early identification of palliative care needs by family physicians: A qualitative study of barriers and facilitators from the perspective of family physicians, community nurses, and patients	Belgium	Qual*, focus groups, interviews	20 GPs, 12 community nurses, 18 people with dementia
		*Aim: explore barriers and facilitators of early identification by family physicians of the palliative care needs*			
de Vries and Drury-Ruddlesden	2019	Advance care planning for people with dementia: Ordinary everyday conversations	New Zealand	Qual*, interviews	23 family caregivers
		*Aim: explore experiences of preparedness and support for family members of people with dementia, before, during and following death.*			
Givens et al.	2018	Advance care planning in community-dwelling patients with dementia	United States	Qual, observations	62 family caregivers
		*Aim: examine aspects of ACP among patients with dementia and health care proxy acceptance of patients’ illness*			
Godwin and Waters	2009	“In solitary confinement”: Planning end-of-life wellbeing with people with advanced dementia, their family and professional carers	United Kingdom	Qual*, observations, interviews	10 people with dementia and family caregivers, 4 wards in nursing home
		*Aim: explore opinions of people with advanced dementia, family and professional carers on good terminal care and well-being*			
Goodman et al.	2013	Preferences and priorities for ongoing and end-of-life care: A qualitative study of older people with dementia resident in care homes	United Kingdom	Qual*, interviews	18 people with dementia, nursing home
		*Aim: explore how older people with dementia discuss priorities and preferences for end-of-life care*			
Goossens et al.	2020a	Shared decision-making in advance care planning for persons with dementia in nursing homes: a cross-sectional study	Belgium	Qual*, Observations	65 wards in nursing home
		*Aim: explore how health professionals and residents with dementia perceive the level of SDM during ACP conversations*			
Goossens et al.	2020b	Improving shared decision-making in advance care planning: Implementation of a cluster randomized staff intervention in dementia care	Belgium	Quant*, Pre-test-post-test CRCT*	311 healthcare professionals, nursing home
		*Aim: examine effects of ACP intervention on SDM for persons with dementia in nursing homes, perceived importance, competence and*			
		*frequency of staff members concerning SDM and facilitating and hindering context elements for the sustainability of the training results*			
Hill et al.	2018	Staff Perspectives of Barriers to Access and Delivery of Palliative Care for Persons With Dementia in Long-Term Care	Canada	Qual*, interviews	22 healthcare professionals, nursing home
		*Aim: investigate experiences of staff delivering palliative care to individuals with dementia to determine how care was delivered, to learn which*			
		*guidelines were used, and whether policies affected the delivery of palliative care*			
Johnson et al.	2009	The communication challenges faced in adopting a palliative care approach in advanced dementia	Australia	Qual*, focus groups, interviews	34 dementia medical experts
		*Aim: examine communication issues affecting implementation of palliative care for persons with dementia in residential aged care facility*			
Karel et al.	2007	Three methods of assessing values for advance care planning: comparing persons with and without dementia	United States	Mixed*, interviews, surveys	176 older adults (of whom 88 people with dementia)
		*Aim: examine the utility of health care values assessment tools for older adults with and without dementia*			
Kayser-Jones	2002	The Experience of Dying: An Ethnographic Nursing Home Study	United States	Qual*, observations, interviews	35 residents, 52 family caregivers: 52, 102 healthcare professionals, nursing home
		*Aim: investigate the process of providing end-of-life care to residents who were dying in nursing homes*			
Morin et al.	2016	Discussing end-of-life issues in nursing homes: a nationwide study in France	France	Quant*, survey	674 family caregivers, nursing home
		*Aim: investigate how discussing end-of-life issues and frequency of conversations associate with care outcomes*			
Poppe et al.	2013	Qualitative Evaluation of Advanced Care Planning in Early Dementia (ACP-ED)	United Kingdom	Qual*, interviews	12 people with dementia, 8 family caregivers, 6 healthcare professionals
		*Aim: explore the acceptability of discussing ACP with people with memory problems and mild dementia shortly after diagnosis*			
Sims-Gould et al.	2010	Care Provider Perspectives on End-of-life Care in Long-Term-Care Homes: Implications for whole-person and palliative care	Canada	Qual*, ethnography	10 residents, nursing home
		*Aim: explore experiences of dying and end-of-life care for persons with dementia in long-term care from the perspective of care providers*			
Sinclair et al.	2016	Consensus views on advance care planning for dementia: a Delphi study	United Kingdom	Delphi study	
		*Aim: investigate consensus views of how ACP should be explained and carried out with people with dementia*			
Sussman et al.	2020	Engaging persons with dementia in advance care planning: Challenges and opportunities	Canada	Qual*, focus groups	10 people with dementia, 8 family caregivers
		*Aim: explore experiences with ACP, concerns related to end-of-life care, and practices supporting positive engagement with ACP*			
Tilburgs et al.	2018	The importance of trust-based relations and a holistic approach in advance care planning with people with dementia in primary care: a qualitative study	Netherlands	Qual*, interviews, focus groups	10 people with dementia, 10 family caregivers, 10 GP’s, 1 focus group (nurses, case managers)
		*Aim: explore barriers and facilitators for ACP with community-dwelling people with dementia*			
van der Steen et al.	2014	White paper defining optimal palliative care in older people with dementia: A Delphi study and recommendations from the European Association for Palliative Care	Worldwide	Delphi study	
		*Aim: define optimal palliative care in dementia*			
Yeh et al.	2021	Improving end-of-life care for persons living with dementia: Bereaved caregivers’ observations and recommendations	United States	Mixed*, surveys interviews	53 family caregivers
		*Aim: elicit recommendations for improving end-of-life care experiences of people with dementia from the perspective of bereaved caregivers*			

*Quant, quantitative research; Qual, qualitative research; Mixed, mixed methods (both qualitative and quantitative research); CRTC, cluster randomized controlled trial.

### Topics

**FIGURE 2:**
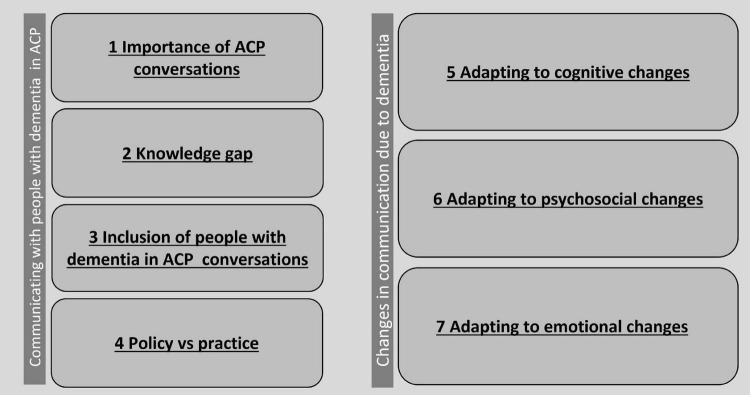
Topics around communication aspects related to dementia and ACP found in the scoping review.

A total of seven topics were identified clustered around two themes. The first four topics reflect on communicating ACP with people with dementia in general (i.e., covering the importance of ACP for people with dementia, the knowledge gap, the inclusion of people with dementia in ACP conversations, and how daily practice may differ from policy). Three more topics emerged around cognitive, psychosocial and emotional changes due to dementia, and how to adapt communication strategies to improve ACP conversations with people with dementia (see Figure 2 for an overview).

#### Topic 1: Importance of Having Advance Care Planning Conversations

All papers underline that ACP is central to high-quality, holistic end-of-life care. Five papers in total specifically focused on the importance of having ACP conversations (Kayser-Jones, 2002; Sims-Gould et al., 2010; Goodman et al., 2013; van der Steen et al., 2014; Sinclair et al., 2016). It is likely to reduce unnecessary interventions, promotes comfort and increases clarity in important decision making (e.g., Goodman et al., 2013). An ethnographic study by Kayser-Jones (2002) showed that “*lack of attention to cultural needs, cognitive status, inadequate staffing, and inappropriate and inadequate communication between healthcare providers and nursing home residents and their families were the predominant factors that influenced the experience of dying*.” The two Delphi studies showed that proactive conversations around care and making decisions are essential for quality of care at the end of life (van der Steen et al., 2014; Sinclair et al., 2016). These conversations should be part of a continuous process of discussing values in life, rather than the single act of completing a form about care preferences (Goodman et al., 2013; Sinclair et al., 2016). To do so, ongoing communication amongst healthcare professionals, people with dementia and family caregivers appears to be essential (Sims-Gould et al., 2010).

#### Topic 2: Knowledge Gap

Although it is clear that communication about care between healthcare professionals and patients is an important determinant of quality end-of-life care, so far, characteristics and determinants of good quality communication have been hard to identify. Two papers described the lack of knowledge on how to provide good quality communication (Goodman et al., 2013; Aasmul et al., 2018). For example, Goodman et al. (2013) interviewed 18 people with dementia about their ACP preferences, and they found it is unclear how people with dementia and their current experiences can inform planning and decision making around ACP. Although numbers of empirical studies on ACP with people with dementia are increasing, it seems that well-powered controlled trials investigating communication between people with dementia and healthcare professionals in long term care facilities (LTCF) are needed (Aasmul et al., 2018).

#### Topic 3: Inclusion of People With Dementia in Advance Care Planning Conversations

Five papers showed that people with dementia are rarely included when discussing ACP (Johnson et al., 2009; Goodman et al., 2013; Poppe et al., 2013; Ampe et al., 2016; Givens et al., 2018). Ampe et al. (2016) clarified that healthcare professionals (1) were more comfortable to talk with family caregivers, and (2) underestimated the capability of people with dementia themselves to talk about preferences related to ACP. However, a decision made by a family caregiver may be inconsistent with the person with dementia their wishes, and family caregivers state making decisions on behalf of someone else around their end-of-life care is difficult (Poppe et al., 2013). Therefore, there is a need to invest in including people with dementia discussing their preferences around ACP.

#### Topic 4: Policy Versus Practice

Only two studies included in this review (Ampe et al., 2016, 2017) show a lack of strategies for implementing ACP conversations in dementia care. Apparently, organizational healthcare policies concerning ACP are present and promising, although often not implemented in daily practice of healthcare professionals. For example, although the intervention “We DECide” by Ampe et al. (2017) positively influenced the intension of performing ACP in participating dementia care units, the actual practice did not improve. Healthcare professionals only managed to involve residents or their family caregivers on a baseline skill level. It is clearly not enough for healthcare professionals to understand and acknowledge evidence-based practices of ACP, and practically oriented training is required to change habits and behaviors. Ampe et al. (2017) suggest adding a strong focus on the organizational context of ACP training (e.g., by including the management of the team in discussions on the topic, or by organizing in service training for trainers).

#### Topic 5: Adapting to Cognitive Changes

A total of eleven papers describe how changes in cognitive abilities with people with dementia may affect the way they participate in ACP, or how to deal with conversation difficulties that arise because of cognitive changes (Karel et al., 2007; Godwin and Waters, 2009; Johnson et al., 2009; Goodman et al., 2013; Poppe et al., 2013; van der Steen et al., 2014; Ampe et al., 2016; Sinclair et al., 2016; Aasmul et al., 2018; Tilburgs et al., 2018; Goossens et al., 2020a). Older people living with dementia show loss of memory, communication, orientation, control, autonomy, independence, self-esteem and relationships (Goodman et al., 2013). Poppe et al. (2013) stated this may affect the way people with dementia make decisions and how they are informed. Johnson et al. (2009) showed that “*dementia has a trajectory that causes a diminishing ability for the person to express their care needs and treatment wishes*.” In their study, Ampe et al. (2016) found that LCTF residents with dementia were no longer capable of discussing important decisions. Moreover, healthcare professionals appear to underestimate communication skills of people with dementia, and their ability to process abstract concepts (Godwin and Waters, 2009). According to van der Steen et al. (2014) communicating with patients and families of people with dementia requires special skills due to cognitive problems that come with dementia complicating decision making. We found several suggestions to overcome these communication problems related to cognitive changes:

##### Question Formulation

After interviewing general practitioners, Tilburgs et al. (2018) suggest ACP should be tailored to the cognitive level of the person with dementia by asking closed instead of open questions. Communication training may help healthcare professionals to remain aware of the way they formulate questions (Aasmul et al., 2018). Moreover, people with dementia should be offered the time to prepare themselves for certain questions and the ability to process them (Goossens et al., 2020a). Apparently, the way questions are asked in ACP conversations should be tailored according to dementia severity. However, interestingly, Karel et al. (2007) found no differences between participants with mild dementia and control participants (without dementia) with regards to question formulation in their survey research. People with mild dementia were able to answer open-ended questions about quality of life and responded to forced-choice questions regarding healthcare values.

##### Timely Initiation

According to Poppe et al. (2013), ACP is usually completed in the last 6 months of life, which may cause problems for people diagnosed with dementia: decision-making capacity and ability to communicate decrease as the disease progresses. Therefore, the initiation of ACP should happen as early in the disease trajectory as possible, preferably shortly after diagnosis, while people with dementia still have the capacity to make informed decisions (Aasmul et al., 2018; Tilburgs et al., 2018), but only when they are ready to do so (Sinclair et al., 2016). Ampe et al. (2016) found that rather than to wait for crisis situations, ACP should be discussed before. They mention admission in a LTCF to be a good moment to talk about ACP. Godwin and Waters (2009) underlined the importance to keep a conversation ongoing; we cannot presume views and conceptions around ACP remain unchanged.

##### Non-verbal Behavior

Several papers underline the importance of observing behavior of people with dementia while in conversation about ACP. Healthcare professionals should not only focus on well formulated (spoken) views (Ampe et al., 2016). If we neglect non-verbal behavior, we may miss important connotations (Godwin and Waters, 2009). Recognition of behaviors as potential signs of discomfort need to be observed and addressed (Johnson et al., 2009).

#### Topic 6: Adapting to Psychosocial Changes

A total of nine papers discussed how psychosocial changes due to dementia may affect ACP conversations or how to overcome difficulties that arise because of these changes (Kayser-Jones, 2002; Karel et al., 2007; Johnson et al., 2009; Goodman et al., 2013; Poppe et al., 2013; Ampe et al., 2016; Aasmul et al., 2018; Tilburgs et al., 2018; Goossens et al., 2020a). Awareness of dementia may affect one’s self-esteem and confidence in a negative way (Goodman et al., 2013). Still, the opportunity to participate in ACP and contribute to choices around their care may help people with dementia feel more empowered (Goossens et al., 2020a). We found several suggestions to improve one’s confidence in ACP conversations:

##### Familiar People

A well-established relationship with a healthcare professional may have a positive effect on the course of an ACP conversation (Ampe et al., 2016). Moreover, follow-up conversations may help to build relationships further, and are also necessary to maintain an ACP routine (Aasmul et al., 2018). However, several papers underline familiarity with the healthcare professional is not as important for successful ACP communication as well-trained and empathetic conversation partners (Karel et al., 2007; Poppe et al., 2013; Tilburgs et al., 2018). According to Karel et al. (2007), familiarity is not essential for good ACP practice. In fact, in their study, non-familiar people who behaved attentive and empathetic, were successful in eliciting care preferences with people with dementia. This suggests more research on the relationship between healthcare professionals and people with dementia is needed.

##### Training of Healthcare Professionals in Effective Communication Skills

A lack of communication skills can be a barrier for conducting successful ACP conversations, such as not listening, trivializing situations and being distant in conversations (Kayser-Jones, 2002; Johnson et al., 2009; Tilburgs et al., 2018; Goossens et al., 2020a). In addition to possessing knowledge about the purpose and goals of ACP, healthcare professionals need to possess effective communication skills in order to initiate and sustain dialog on the future care of people with dementia (Johnson et al., 2009; Poppe et al., 2013). In a study by Poppe et al. (2013), healthcare professionals identified knowledge about dementia, available resources and knowledge of one’s own limitations as key skills and competencies for discussing ACP. Feeling confident when discussing ACP was mentioned to be important and healthcare professionals found having experience in dealing with difficult conversations increased their confidence.

##### Healthcare Professionals Empathy

Discussing their findings of their study on improving ACP communication between healthcare professionals and people with dementia, Aasmul et al. (2018) stated that a low level of empathy with staff may have affected ACP conversations in a negative way. This fits well with work of Tilburgs et al. (2018), who found non-empathic attitudes by general practitioners to be a barrier of ACP with people with dementia. It seems to be important for healthcare professionals to have an empathetic attitude while talking to people with dementia about ACP.

#### Topic 7: Adapting to Emotional Changes

Dementia causes substantial changes in one’s life that may elicit different emotions and feelings compared to feelings people had before their diagnosis, or compared to those with other diseases. A total of ten papers reported on how emotional changes that come with dementia may possibly affect ACP conversations, or on how to overcome challenges due to these changes (Kayser-Jones, 2002; Godwin and Waters, 2009; Poppe et al., 2013; Beernaert et al., 2014; Ampe et al., 2016; Aasmul et al., 2018; Givens et al., 2018; Hill et al., 2018; de Vries and Drury-Ruddlesden, 2019; Yeh et al., 2021). People with dementia often experience agitation, fear, confusion, and pain. This may be difficult to cope with, for themselves or those around them (Hill et al., 2018). The thought of needing palliative care can be confronting, and therefore ACP conversations tend to be avoided by some (Beernaert et al., 2014). In general, ACP conversations are practically oriented, and not so much focusing on emotions. Although crisis situations often contain emotional moments, there may be little room for discussing emotions when making decisions, possibly related to time pressure. This could be a missed opportunity to understand which and how emotions influence certain decisions (Ampe et al., 2016). We found several suggestions to cope with emotions better in ACP conversations:

##### Not Avoiding the Topic

Often, in day-to-day conversations between people with dementia and healthcare professionals, safe topics and social chit-chat are most apparent (Kayser-Jones, 2002). Beernaert et al. (2014) found that neither people with dementia nor healthcare professionals initiate discussion of non-acute care needs in day-to-day conversations. According to Godwin and Waters (2009), the ability and especially the willingness of people with dementia to talk about abstract concepts such as death is underestimated with healthcare professionals. Yeh et al. (2021) underline that initiative for ACP conversations between people with dementia and healthcare professionals can also come from family caregivers.

##### Regular Meetings

It may be beneficial for healthcare professionals to initiate regular conversations with people with dementia to talk about ACP. Aasmul et al. (2018) suggested quarterly meetings with nursing home residents with dementia and monthly meetings with their family caregivers. According to a study in nursing homes by Ampe et al. (2016), people with dementia should be engaged actively in the ACP process, not only when they are admitted to a LTCF, “but throughout their stay.” They state it is the responsibility of healthcare professionals to stimulate colleagues and other care workers to pick up on conversations and communicate relevant information about ACP.

##### Normalizing Advance Care Planning

Conversations around important topics such as end-of-life and care preferences may cause some anxiety in patients, but there is a need to normalize these kind of conversations. Poppe et al. (2013) showed that ACP conversations gave people with dementia time to think about their future. Some were relieved and less worried after discussing their preferences, and they felt supported by their family and services. It was important for them that family caregivers and healthcare professionals were attentive to their preferences. Moreover, discussing ACP within families makes room for having those conversations in an informal, day-to-day setting (de Vries and Drury-Ruddlesden, 2019). According to Givens et al. (2018), ACP conversations in family settings do not happen enough but would benefit everyone involved.

## Discussion

With this scoping review, we mapped existing literature on communication aspects related to dementia in ACP conversations. Seven topics (importance of having ACP conversations, knowledge gap, inclusion of people with dementia in ACP conversations, policy vs. practice, adapting to cognitive changes, adapting to psychosocial changes, and adapting to emotional changes) emerged. Practical suggestions for healthcare professionals to improve ACP communication are provided and summarized in Box 1. This review underlines the scarcity of studies focusing on “how” to talk to a person with dementia about their ACP preferences; more research is needed on non-verbal communicative aspects of ACP conversations, timing and implementation of these conversations, and preferences of individuals with dementia related to these conversations, as results of studies so far have been inconsistent or have not given any or little attention to these topics.

**BOX 1. Practical suggestions for healthcare professionals to improve ACP communication.**

• Make continuous and active conversations part of the ACP practice, rather than the single act of completing a form about care preferences.

• Make an effort to include people with dementia themselves in ACP conversations.

• Tailor the questions asked in ACP conversations according to dementia severity and personal needs.

• Initiate ACP as early in the disease trajectory as possible, preferably shortly afterward the diagnosis.

• Focus not only on well formulated (spoken) views but also pay attention to non-verbal behavior of person with dementia.

• There needs to be empathy and attentive listening.

• Do not underestimate the ability and the willingness of people with dementia to talk about abstract concepts such as ACP.

• Initiate ACP conversations on a regular basis.

• Normalize talking about ACP by initiating ACP conversations in informal settings.

One topic that was mentioned in the literature several times but seems to lack empirical evidence, is how expressive behavior related to dementia may affect the process of an ACP conversation between a patient and a healthcare professional. Research underlines the importance of reading non-verbal behavior for ACP conversation processes and outcomes (Godwin and Waters, 2009; Johnson et al., 2009; Ampe et al., 2016), but how to do so is still to be studied. Numerous studies show changes in expressive abilities of people with dementia [e.g., emotional expressions, review by Lee et al. (2019); expressions of apathy, Kumfor et al., 2018; and mutual eye gaze, Sturm et al., 2011], and may be extra prone to affect behavior in conversations around sensitive topics of ACP.

Another important topic that needs more research is timing and implementation of ACP conversations and practice. Research seems to underline the significance of “early” initiated conversations, and that initiation in care facilities may be too late to include people with dementia (Poppe et al., 2013; Ampe et al., 2016; Sinclair et al., 2016; Aasmul et al., 2018; Tilburgs et al., 2018). More research is needed on how to define the right moment. Several papers suggest healthcare professionals to “have a continuous conversation” with people with dementia about ACP, and to “talk about ACP in an informal way and often.” However, it seems to be difficult to implement such suggestions in practice. Healthcare professionals often feel incapable of initiating conversations on sensitive topics like ACP and tend to avoid these (Beernaert et al., 2014). Future research should have a strong focus on how to implement ACP in practice, rather than only formulating ACP policies. Also, although our findings suggest that ACP conversations are needed on a regular base, and healthcare professionals investing time seems necessary, the ideal duration of an ACP conversation remains unclear (e.g., de Vries and Drury-Ruddlesden, 2019).

Further research is needed on the optimal timing and frequency of conversations, but also on models of care in which healthcare professionals who are best positioned to conduct and personalize ACP, are supported to do so. A review by Piers et al. (2018), describes evidence-based guidelines for healthcare professionals across settings in the practical application of ACP in dementia care. Amongst recommendations on topics such as documentation and end-of-life decisions, limited suggestions are made around communication strategies in ACP conversations that are in line with the recommendations as presented in Box 1(e.g., adjust one’s communication style and content to their own level and rhythm). Still, papers included in this scoping review showed different findings with regard to the importance of familiar healthcare professionals leading ACP conversations, the effect of (lack of) empathy by the healthcare professional, and whether or when an approach should be more or less directive. Preferences for the way an ACP conversation is held may be personal, and can change over time, depending on many factors, such as cultural background or even mental conditions. For example, in some cultures, fostering autonomy of the person with dementia is important, while in other cultures, a paternalistic approach may be useful at times. As this is barely reflected in the studies included in this scoping review, future work needs to focus on personal preferences that demand a level of flexibility of healthcare professionals to adopt different approaches as needed.

This scoping review has several strengths and limitations. Our strength is that we focused specifically on communicative aspects of ACP conversations with people with dementia, uncovering significant gaps in literature and providing recommendations for research and practice. Unfortunately, most studies covered this topic within a broader scope, focusing on ACP practice related to dementia in general, leaving several important factors underexposed. No papers that were included in the scoping review focused on cultural differences as a determinant for how to communicate ACP with people with dementia, limiting the generalizability while also exposing a need for future studies on ACP to include non-Western populations. In fact, all studies included were conducted in Western high-income studies. A reason for this could be we only included papers written in English. Another complicating matter when interpreting results and recommendations of the papers included in the scoping review, was that papers referred to different ACP policies that were custom for specific countries. Legislation and guidelines at national and international level may determine how ACP is approached and is still being developed (Alzheimer Europe, 2020). Still, scoping the literature, we did find a number of practical implications for ACP practice in general, to be adapted by healthcare practitioners and family caregivers, in order to improve ACP practices. Conversations about ACP should be continuous practice, with an active role for people with dementia themselves. Timing, content and form have to be tailored to their cognitive, psychosocial and emotional needs. This study underlines the need for more thorough, empirical studies that look into how to talk to people with dementia about future care.

## Data Availability Statement

The original contributions presented in the study are included in the article/supplementary material, further inquiries can be directed to the corresponding author/s.

## Author Contributions

MV, HS, JS, and DP: conceptualization. MV and HS: data curation. MV and JS: formal analysis. MV: funding acquisition and writing—original draft. HS, JS, and DP: writing—review and editing. All authors approved the final version to be published and read and agreed to the published version of the manuscript.

## Conflict of Interest

The authors declare that the research was conducted in the absence of any commercial or financial relationships that could be construed as a potential conflict of interest.

## Publisher’s Note

All claims expressed in this article are solely those of the authors and do not necessarily represent those of their affiliated organizations, or those of the publisher, the editors and the reviewers. Any product that may be evaluated in this article, or claim that may be made by its manufacturer, is not guaranteed or endorsed by the publisher.
